# Transcriptomic Profiling of MicroRNA and Non-Coding RNA from Whole Blood of African Americans with MASLD

**DOI:** 10.3390/ijms27041666

**Published:** 2026-02-09

**Authors:** Tanmoy Mondal, Brent E. Korba, Christopher A. Loffredo, Coleman I. Smith, Ruth Quartey, Jasneet Sahota, Gemeyel Moses, Charles D. Howell, Gail Nunlee-Bland, Zaki A. Sherif, Somiranjan Ghosh

**Affiliations:** 1 Department of Biology, Howard University, Washington, DC 20059, USA; tanmoy.mondal@howard.edu (T.M.); brentkorba@gmail.com (B.E.K.); jasneet.sahota@bison.howard.edu (J.S.); mosesga@alumni.vcu.edu (G.M.); 2 Department of Oncology, Georgetown University, Washington, DC 20057, USA; cal9@georgetown.edu; 3 MedStar Georgetown Transplant Institute, Georgetown University School of Medicine, Washington, DC 20007, USA; coleman.i.smith@gunet.georgetown.edu; 4 Department of Internal Medicine, College of Medicine, Howard University, Washington, DC 20059, USA; rquartey@gmail.com (R.Q.); charles.howell@howard.edu (C.D.H.); 5 Departments of Pediatrics and Child Health, College of Medicine, Howard University, Washington, DC 20059, USA; gnunlee-bland@howard.edu; 6 Departments of Biochemistry and Molecular Biology, College of Medicine, Howard University, Washington, DC 20059, USA; zaki.sherif@howard.edu

**Keywords:** microRNAs (miRNAs), non-coding RNAs (ncRNAs), MASLD, African Americans, transcriptomics

## Abstract

Metabolic dysfunction-associated steatotic liver disease (MASLD), formerly known as non-alcoholic fatty liver disease (NAFLD), is a growing health concern, yet the role of non-coding RNAs (ncRNAs), including microRNAs (miRNAs), in its pathogenesis remains poorly understood. In this pilot study, we aimed to identify significantly expressed miRNAs and ncRNAs and correlate transcriptomic patterns of the findings with previously identified coding gene expression profiles to explore potential regulatory mechanisms in MASLD. Participants were selected from an existing study population. We conducted transcriptomic profiling of miRNAs and other ncRNAs in whole-blood samples from African American (AA) individuals with MASLD and matched controls (*n* = 4 per group) as a discovery cohort. A subsequent qRT-PCR validation study was performed in 30 participants, including 14 individuals with MASLD and 16 controls. miRNA sequencing was performed by Zymo, USA, followed by miRNA extraction using the Zymo-Seq™ miRNA Library Kit. Differentially expressed miRNAs and ncRNAs were analyzed using Ingenuity Pathway Analysis (IPA) to identify associated biological pathways. A total of 1412 miRNAs and 5423 other ncRNAs were identified in this study. Among them, 35 miRNAs and 28 other ncRNAs exhibited significant differential expressions (fold-change cutoff 1.5, *p* < 0.05). miR-206 was consistently upregulated, whereas miR-1343-5p, miR-1299, miR-224-5p, and miR-193a-5p were downregulated across all samples. miR-206 upregulation and miR-185-3p/miR-224-5p/miR-218-5p downregulation were validated, associating with lipid metabolism impairment and hepatic fibrosis via the AMPK/TGF-β pathway, implicating ncRNA-mediated regulation. To our knowledge, this is the first whole-blood non-coding RNA transcriptomic study in AA MASLD, an under-represented population. This small-scale pilot study requires validation in large multi-ethnic cohorts to confirm generalizability.

## 1. Introduction

Metabolic dysfunction-associated steatotic liver disease (MASLD), formerly known as non-alcoholic fatty liver disease (NAFLD), is now recognized as the most prevalent chronic liver disease globally, affecting approximately 25–30% of the adult population [1]. The disease spectrum ranges from simple hepatic steatosis (fat accumulation in the liver) to non-alcoholic steatohepatitis (NASH), which can further progress to advanced fibrosis, cirrhosis, and hepatocellular carcinoma (HCC) [2]. MASLD is strongly linked to metabolic comorbidities, including obesity, insulin resistance, type 2 diabetes mellitus (T2DM), hypertension, and dyslipidemia. Importantly, MASLD is not just a liver-centric disease but is increasingly recognized as a hepatic manifestation of systemic metabolic dysfunction [3]. The recent transition from NAFLD to MASLD reflects an evolving understanding that metabolic dysfunction is central to the disease’s pathogenesis, irrespective of alcohol consumption [4]. Unlike previous definitions that excluded other liver conditions primarily based on alcohol thresholds, MASLD emphasizes the metabolic underpinnings, including insulin resistance, visceral adiposity, and dysregulated lipid metabolism as key drivers of disease progression [4].

Several pathophysiological mechanisms contribute to MASLD development, including hepatic lipid accumulation, mitochondrial dysfunction, oxidative stress, endoplasmic reticulum stress, chronic low-grade inflammation, and fibrogenesis [5]. Insulin resistance plays a pivotal role by increasing free fatty acid flux to the liver, promoting de novo lipogenesis, and impairing lipid exports. This lipid overload leads to lipotoxicity, which triggers hepatocellular injury and activation of hepatic stellate cells, culminating in fibrosis [6]. Despite its high prevalence, MASLD exhibits considerable heterogeneity across all populations. African Americans (AAs) exhibit paradoxical MASLD features, characterized by relatively lower hepatic steatosis despite a high burden of metabolic risk factors, including obesity, insulin resistance, and type 2 diabetes [7,8,9]. While genetic and environmental contributors to ethnic differences in MASLD have been proposed, the contribution of miRNAs and other non-coding RNAs to these population-specific disease patterns has not been systematically investigated.

A limited, but increasing, number of studies have underscored the regulatory roles of non-coding RNAs (ncRNAs) and microRNAs (miRNAs), in the development and progression of MASLD [10,11]. Importantly, the majority of prior non-coding RNA studies in MASLD have been conducted predominantly in Caucasian or European cohorts and have focused largely on hepatic tissue, serum, or plasma samples. Whole-blood transcriptomic profiling of miRNAs and other non-coding RNAs in AAs with MASLD is notably absent from the current literature. This lack of representation limits the development of ancestry-informed biomarkers and constrains our understanding of race-specific regulatory mechanisms that may influence disease susceptibility, progression, and clinical outcomes.

miRNAs are small non-coding RNA molecules that post-transcriptionally regulate gene expression by targeting mRNAs for degradation or translational repression. Small, non-coding RNA molecules also play a crucial role in regulating gene expression by targeting mRNA transcripts [10] via binding to the 3′ untranslated region (3′UTR) of target mRNAs, leading to mRNA degradation or inhibition of translation. This post-transcriptional regulation of gene expression helps control various cellular processes, including cell growth, development, and differentiation [10]. 

miRNAs play crucial roles both within cells and in the extracellular environment by regulating gene expression intracellularly and functioning as intercellular messengers when secreted into extracellular fluids. They are transported via exosomes or RNA-binding protein complexes such as those involving AGO proteins to modulate gene expression in target cells [11]. Furthermore, an expanding body of evidence demonstrates that ncRNAs operate within competitive endogenous RNA (ceRNA) networks, where lncRNAs or circular RNAs (circRNAs) act as molecular sponges, sequestering specific miRNAs and thereby modulating their availability and function. For example, circ_0057558 and lncRNA MALAT1 have been shown to promote hepatic lipid accumulation and steatosis by sponging miR-206, a key regulator of lipid metabolism and insulin sensitivity [12,13]. Similarly, lncRNAs such as LINC00963 and LINC01234 are implicated in liver fibrosis by modulating fibrogenic signaling through ceRNA mechanisms [14,15]. However, there have been few attempts to coordinate the expression patterns of non-coding RNAs with the altered gene expression patterns in MASLD subjects, especially within the same individuals.

To determine the feasibility of addressing this issue, a case-control pilot study of the expression patterns of ncRNAs was conducted using individuals from a previous [8,9] transcriptomic investigation conducted by our group. This population was comprised of AAs to address the lack of inclusion of minority participants in prior studies of transcriptomic patterns in MASLD patients. This study utilized whole-blood samples, which we and others have demonstrated to have substantial utility in establishing transcriptomic patterns that largely overlap the patterns observed in hepatic tissue [8,9]. The current pilot study, to the best of our knowledge, reports for the first time the transcriptomic profiling study of miRNA and other ncRNA expressions in whole-blood samples from AA individuals with MASLD. Accordingly, the objectives of this pilot study were to (1) identify differentially expressed miRNAs and other non-coding RNAs in whole blood from AA individuals with early-stage MASLD; (2) validate key dysregulated miRNAs using qRT-PCR in an independent cohort; and (3) elucidate the associations between identified ncRNA signatures and biological pathways related to hepatic fibrosis and lipid metabolism. By addressing these aims, this study seeks to establish a foundational framework for race-specific, non-invasive biomarker discovery in MASLD.

## 2. Results

### 2.1. Study Participants

All selected participants (total *n* = 38) were AAs and were part of the previous transcriptomic study from the Washington DC area [8]. The participants were separated into two groups: a control group of individuals without MASLD and a case group of individuals with early-stage MASLD (confirmed hepatic steatosis and exhibiting one or more comorbid metabolic features, viz., type 2 diabetes, hypertension, hyperlipidemia, or obesity). A qRT-PCR validation study was then performed in 30 participants, including 14 from the MASLD group and 16 from the control group. MASLD was diagnosed based on standard criteria, including confirmed hepatic steatosis (based on their imaging/biopsy records supported by the presence of hepatic steatosis on cross-sectional imaging, liver elastography, and/or histological confirmation by percutaneous liver biopsy) and presence of one or more comorbid metabolic features, viz., type 2 diabetes, hypertension, hyperlipidemia, or obesity [8]. Table 1 displays the characteristics of each group. No significant differences in age, BMI, or HbA1c were observed in the discovery group; however, a significant difference (*p*-value 0.01) in HbA1c (%) was observed in the validation cohort, with mean HbA1c levels of 5.35 ± 0.57% in the control group and 6.58 ± 1.56% in the MASLD group. For the purposes of liver steatosis staging, we used the S grade (S0–S3); the higher the grade, the higher the percentage of liver affected by fatty changes (Table 2).

### 2.2. miRNA and ncRNA Sequencing, Differential Expression, and Number of Reported Studies

**Figure 1:**
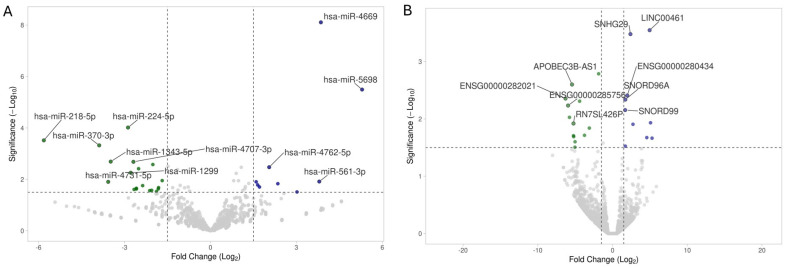
Transcriptomic profiling of miRNA and other ncRNAs from whole blood. (**A**) Volcano plot showing differential expression of microRNA (log2 of fold change; x-axis) and statistical significance of this change (−log10 of significance; y-axis) for MASLD cases compared to the control group. (**B**) Volcano plot showing differential expression of other ncRNAs (log2 of fold-change; x-axis) and statistical significance of this change (−log10 of significance; y-axis). Colored points represent differentially expressed miRNAs and other ncRNAs (cutoff FDR 0.05) with a magnitude of change = 1.5 for those that are either overexpressed (blue; fold change ≥ 1.5, FDR < 0.05) or under expressed (green; fold change ≤ −1.5, FDR < 0.05). The most significant are labeled.

Out of a total of 1412 miRNA identified transcripts, 35 miRNAs in the MASLD cases were significantly differentially expressed when compared to the controls (fold-change cutoff 1.5-fold and *p*-value < 0.05), with 24 downregulated and 11 upregulated (Figure 1A and Table 3). Out of a total of 5423 other ncRNAs transcripts, 28 were significantly differentially expressed, with 17 downregulated and 11 upregulated in the MASLD cases compared to the controls (Figure 1B and Table 4).

**Figure 2:**
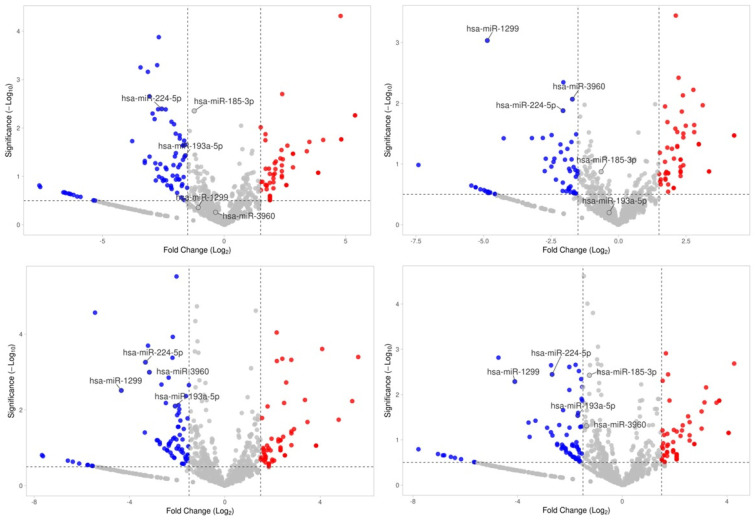
Volcano plot showing differential expression of miRNAs (log2 of fold change; *x*-axis) and statistical significance of this change (−log10 of significance; y-axis) for each MASLD case compared to the control group. Colored points represent differentially expressed miRNAs with a magnitude of change = 1.5 for those that are either overexpressed (red; fold change ≥ 1.5, FDR < 0.05) or under expressed (blue; fold change ≤ −1.5, FDR < 0.05). Common miRNAs are labeled.

We also examined the differential miRNA expression of each individual case compared to the control group’s expression status (Figure 2). We observed that miR-1299, miR-193a-5p, miR-185-3p, miR-3960, miR-1343-5p, and miR-224-5p were significantly downregulated and miR-206 was significantly upregulated in all the MASLD subjects.

### 2.3. Top Biofunctions, Canonical Pathways, and Network Analysis

**Figure 3:**
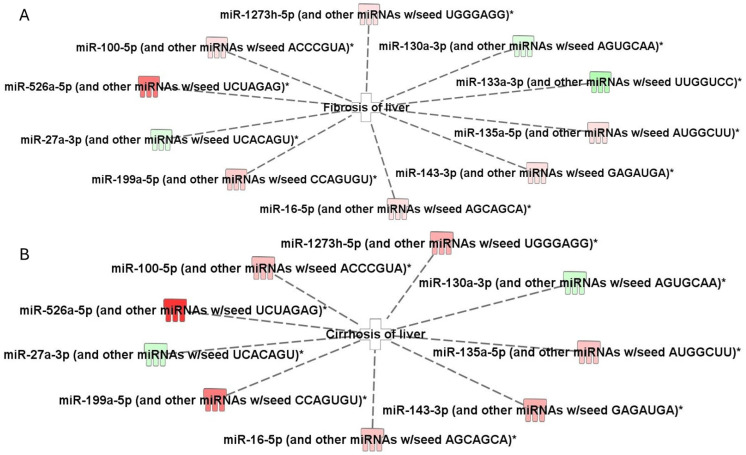
Ingenuity Pathway Analysis (IPA)-identified differentially expressed miRNAs connected to fibrosis and cirrhosis of the liver. (**A**) Differentially expressed miRNAs connected with fibrosis. (**B**) Differentially expressed miRNA connected with cirrhosis. miRNAs that are upregulated are represented in red, while downregulated miRNAs are depicted in green. The intensity of the red and green colors reflects the degree of up- or downregulation, respectively, in the expression dataset. * Grouped with other miRNAs that share the same seed sequence.

The IPA revealed significant biofunctions, including “fibrosis of liver” and “cirrhosis of liver,” with significant overlap percentages (*p*-value < 0.05). These biofunctions were associated with 10 distinct miRNAs: miR-100-5p, miR-1273h-5p, miR-130a-3p, miR-133a-3p, miR-135a-5p, miR-143-3p, miR-16-5p, miR-199a-5p, miR-27a-3p, and miR-526a-5p (Figure 3A,B).

**Figure 4:**
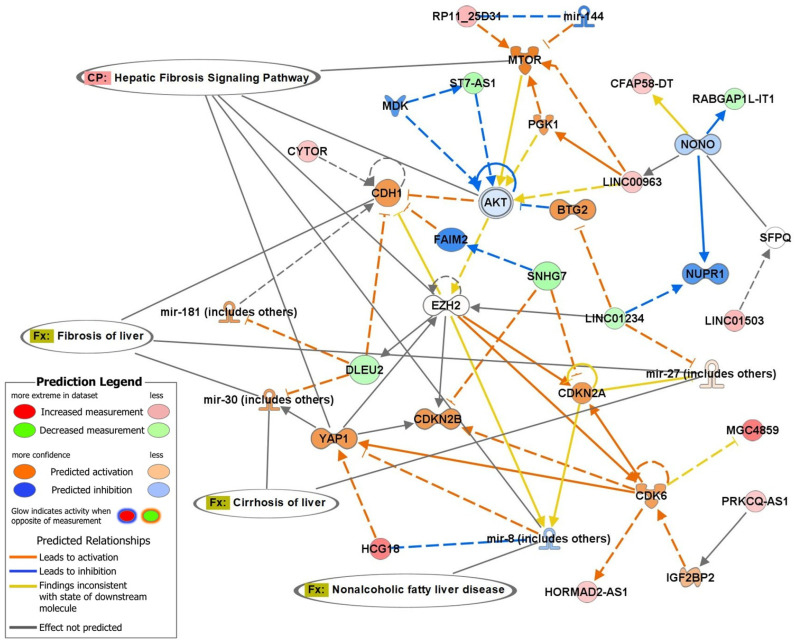
Network of other differentially expressed (*p* < 0.05) ncRNAs in the MASLD participants, relative to control group. The network was generated using Ingenuity Pathway Analysis (IPA) from QIAGEN, USA. In the figure, red arrows = activation, and blue arrows = inhibition.

In addition to the miRNA findings, other ncRNAs were prominently featured in the network analysis (Figure 3). Several long non-coding RNAs (lncRNAs), such as *LINC00963*, *SNHG7*, *CYTOR*, and *HORMAD2-AS1*, were identified as key regulators in the hepatic fibrosis and cirrhosis pathways. These lncRNAs interacted with critical molecular hubs, including *EZH2, AKT,* and *YAP1*, highlighting their regulatory roles in fibrosis-related processes. The network further identified ncRNAs, such as *RP11* and *LINC01234*, as contributing to the modulation of gene expression, emphasizing their potential involvement in liver disease pathogenesis. The canonical pathway analysis (Figure 4) highlighted the “hepatic fibrosis signaling pathway” as a central mechanism linking these ncRNAs and miRNAs to critical molecular and cellular functions. The interactions between miRNAs, lncRNAs, and target genes suggest a tightly regulated network underlying MASLD (Supplementary Table S1).

### 2.4. qRT-PCR Validation of Differentially Expressed miRNAs

**Figure 5:**
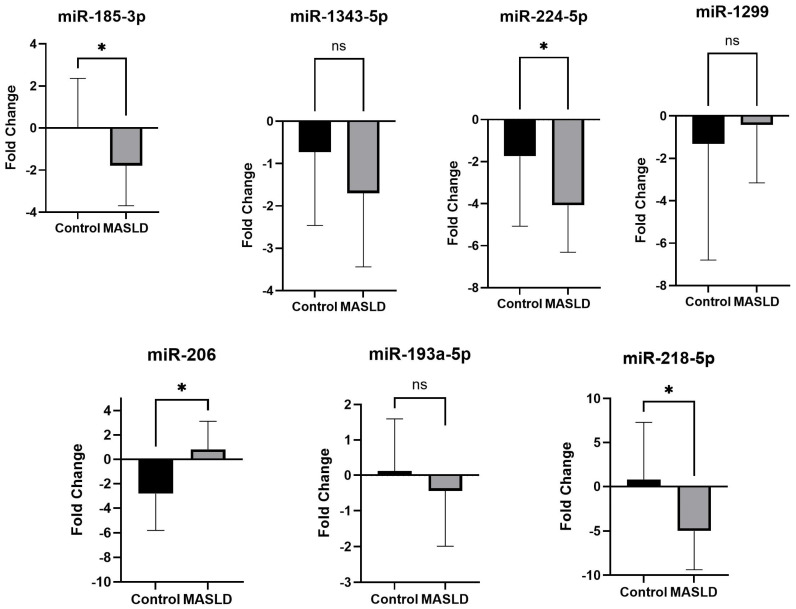
Quantitative real-time PCR (qRT-PCR) of major miRNAs that were significantly up- or downregulated in all MASLD samples (observed by global miRNA assay) compared to the control. For the validation study, we included 30 additional participants (14 from the MASLD group and 16 from the control group), and we used the Applied Biosystem^®^ pre-configured TaqMan array card (96 well array card format) (Thermo Fisher Scientific, Foster City, CA, USA) to examine the expression of the target of interest, viz., 18s (manufacturing control), GAPDH (internal control), miR-206, miR-1343-5p, miR-224-5p, miR1299, miR-193a-5p, miR185-3p, and miR-218-5p, as identified based on the previous data. We selected only those patients who were at the early stages of development of MASLD (confirmed hepatic steatosis and exhibiting one or more comorbid metabolic features, viz., type 2 diabetes, hypertension, hyperlipidemia, or obesity). Six samples per plate were analyzed by High-Throughput FAST Real-Time PCR (Applied Biosystems, Foster City, CA, USA). qRT-PCR was carried out on a QuantStudio 7 Pro PCR system (Applied Biosystems, Foster City, CA, USA). Significance was determined using unpaired *t*-tests (* *p* < 0.01), and results are presented as the mean ± SEM from triplicate experiments. “ns” means not significant.

To validate the sequencing-derived miRNA signature, we performed qRT-PCR in 30 participants (MASLD: *n* = 14; controls: *n* = 16) who were not part of the above profiling experiments. We targeted seven miRNAs that were consistently dysregulated across all MASLD subjects in the discovery dataset. As shown in Figure 5, the qRT-PCR results demonstrated strong agreement with the discovery data, both in fold-change direction and level of significance. miR-206, which showed an average fold change of 0.822 ± 2.29 in validation dataset and 2.22 ± 0.19 in the discovery dataset, was significantly upregulated in MASLD (*p* < 0.05). Among the downregulated miRNAs, miR-185-3p (–1.78 ± 1.90), miR-224-5p (–4.07 ± 2.24), and miR-218-5p (–5.01± 4.36) demonstrated significant reductions in MASLD (*p* < 0.05). Three additional miRNAs—miR-1343-5p (–0.74 ± 1.94), miR-1299 (–0.42 ± 2.73), and miR-193a-5p (–0.43 ± 1.55)—displayed consistent downward trends compared to controls, although these did not reach statistical significance in the validation cohort, likely reflecting biological variability and the modest sample size (Figure 5 and Supplementary Table S3). Notably, miR-1343-5p and miR-1299 demonstrated directionally consistent downregulation across both discovery and validation datasets (*n* = 30), suggesting that limited statistical power and AA-specific immunometabolic contexts may contribute to the absence of statistical significance rather than a lack of biological relevance.

## 3. Discussion

In this pilot study, we present a pilot transcriptomic analysis of miRNA and other ncRNA expression profiles in whole-blood samples from AA individuals with early-stage MASLD. By identifying differentially expressed ncRNA species, our work aims to generate hypotheses on regulatory pathways involved in the early stage of MASLD.

Integrating sequencing data with qRT-PCR validation, four miRNAs, miR-206, miR-185-3p, miR-224-5p, and miR-218-5p, demonstrated statistically significant dysregulation, highlighting their strong involvement in lipid metabolism, inflammation, and fibrogenesis in MASLD (Table 5). In the present study, miR-206 was consistently upregulated in whole blood from all MASLD participants in both the discovery and validation cohorts, indicating a robust and reproducible circulating signature associated with early-stage disease. miR-206, which plays a dual role in lipid metabolism and insulin sensitivity, has been shown to inhibit hepatic lipogenesis and gluconeogenesis, thereby promoting insulin responsiveness [23]. We observed miR-206 upregulation, and prior mechanistic studies provide biological plausibility for a compensatory or protective regulatory response in MASLD. Chen et al., 2021, demonstrated that circ_0057558 promotes hepatic lipid accumulation by targeting miR-206, which, in turn, regulates the ROCK1/AMPK signaling pathway [12]. The elevated miR-206 levels observed in our AA cohort may favor AMPK activation, potentially limiting hepatic lipid accumulation and fibrogenic progression. This integrated interpretation may help explain the paradoxical phenotype reported in AAs, characterized by lower hepatic steatosis despite a high burden of metabolic risk factors [12]. Xiang et al., 2022, reported that miR-206 negatively regulates ARNT expression, impacting the PPARα/CD36 pathway, which plays a crucial role in hepatic lipid metabolism, and showed that manipulating miR-206 levels alters lipid accumulation and liver injury severity [13]. Mohammed et al., 2024, identified elevated circulating miR-206 levels in patients with hepatic steatosis and hyperlipidemia, suggesting a systemic role for miR-206 in metabolic regulation, possibly as a compensatory response to metabolic dysfunction [60]. In light of these findings, the consistent upregulation (2.5-fold) of miR-206 in MASLD patients suggests a compensatory role in mitigating hepatic insulin resistance and lipid accumulation, aligning with previous findings demonstrating its regulatory effect on the AMPK and PPARα pathways [12,13].

miR-224-5p is known to be involved in multiple regulatory processes such as lipid accumulation, endoplasmic reticulum stress, mitochondrial damage, inflammatory response, autophagy, and hepatic stellate cell activation, potentially influencing the progression of MASLD [61,62]. Upregulated miR-224-5p targets the leptin (LEP) gene and leads to its suppression through dysregulation of the AMPK pathway, which is associated with MASLD progression [18]. Intriguingly, miR-224-5p exhibited significant downregulation in both discovery and validation datasets (–4.07 ± 2.24; *p* < 0.05), despite prior studies reporting upregulation in non-AA MASLD populations [61,63]. Given that most published MASLD miRNA studies have profiled hepatic tissue or serum/plasma rather than whole blood, differences in biological compartment may partially explain this discordance. Directionality of miRNA dysregulation is not uniformly conserved between liver tissue and peripheral blood, reflecting differences in cellular origin, immune composition, and systemic metabolic state. Indeed, non-MASLD studies have reported context-dependent and compartment-specific regulation of miR-224-5p, with opposing patterns observed between tissue and circulating immune cells. Nevertheless, the consistent downregulation of miR-224-5p across all AA MASLD participants in this study suggests that circulating miRNA signatures may capture regulatory processes distinct from hepatic tissue expression and potentially shaped by ancestry-associated immunometabolic environments [7,64]. Additionally, dysregulation of other miRNAs, such as miR-370-3p and miR-218-5p, further connects pathways linked to lipid metabolism, inflammation, and hepatic fibrosis, underscoring their multifactorial contribution to MASLD pathobiology [16,17]. Both miR-185-3p and miR-218-5p were significantly downregulated by qRT-PCR (*p* < 0.05) and have regulatory roles in NF-κB-mediated inflammation and hepatic lipogenesis. Future studies involving diverse multi-ethnic cohorts are essential to validate whether the regulatory patterns of miR-224-5p are indeed influenced by racial or genetic backgrounds and to elucidate potential AA-specific mechanisms.

Downregulation of miR-1343-5p and miR-1299, both known negative regulators of TGF-β and Wnt/β-catenin signaling, respectively, may exacerbate fibrogenesis by promoting hepatic stellate cell activation and extracellular matrix deposition [14,15,19]. We observed the downregulation of miR-1343-5p in all four MASLD patients in our study, supporting our previously published findings, which noted an upregulation of TGF-β and activation of the hepatic fibrosis signaling pathway in these subjects [8]. Another previous study reported that upregulation of circulating miR-1343-5p is a potential biomarker in MASLD in adolescents with severe obesity [65]. Although miR-1343-5p (–0.74 ± 1.94) and miR-1299 (–0.42 ± 2.73) showed consistent downward trends across discovery and validation, these changes did not reach statistical significance in the validation cohort, likely reflecting biological variability.

**Figure 6:**
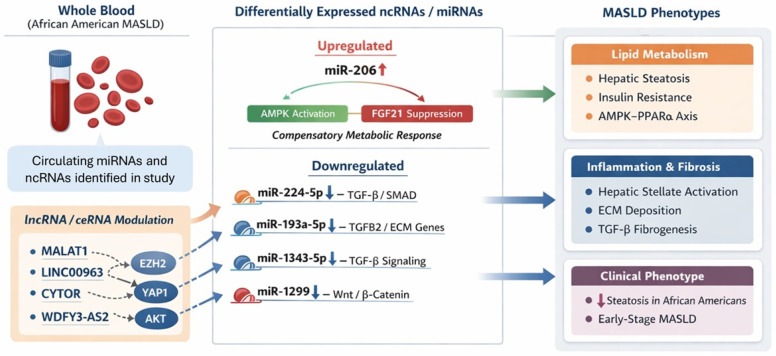
Schematic representation of the proposed regulatory network linking differentially expressed circulating miRNAs and non-coding RNAs identified in this study to key metabolic and fibrotic pathways involved in MASLD pathogenesis. Upregulation of miR-206 may reflect a compensatory response promoting AMPK-associated lipid metabolic regulation, whereas downregulation of miR-224-5p, miR-193a-5p, miR-1343-5p, and miR-1299 may enhance TGF-β- and Wnt/β-catenin-mediated fibrogenic signaling. Long non-coding RNAs (e.g., MALAT1, LINC00963, and CYTOR) may further modulate these effects through ceRNA interactions, collectively contributing to lipid dysregulation, inflammation, and fibrosis in early-stage MASLD.

The significant downregulation of miR-193a-5p in the discovery cohort study, which has been previously reported as a biomarker for liver fibrosis and cirrhosis [66,67,68,69,70], reinforces its potential role as an early indicator of MASLD. The downregulation of miR-193a-5p is thought to inhibit pro-fibrotic gene targets, such as TGFB2, thereby promoting hepatic stellate cell activation, a central event in the fibrotic cascade [71]. This observation suggests a plausible link between miR-193a-5p downregulation and progressive fibrogenesis in MASLD, highlighting its relevance in the early stages of disease pathogenesis (Figure 6). Moreover, given that miR-193a-5p plays a regulatory role in extracellular matrix remodeling and fibrotic signaling, its dysregulation may contribute directly to the hepatic tissue alterations characteristic of MASLD progression [72]. Notably, we observed a similar downregulation trend in the validation study, but the relationship was not significant. Our prior research identified dysregulation of the TGFB1 and E2F1 genes and associated pathways in peripheral blood samples in the cohort of AA patients with early-stage MASLD [8] used for the current study. We observed the activation of hepatic fibrosis signaling pathways and their potential role in the development of hepatocellular carcinoma, particularly when TGFB1 was upregulated and E2F1 was downregulated [8]. However, the study did not establish a definitive role for TGFB1 and E2F1 regulation in the development of hepatic steatosis or the lower prevalence of MASLD in AAs. Some previous studies have reported an upregulation trend of miR-193a-5p in blood serum and plasma samples, mainly in Caucasian populations [67,68].

In addition to miRNAs, our pathway and network analyses identified several lncRNAs involved in fibrosis-related signaling networks. lncRNAs such as LINC00963, SNHG7, CYTOR, and HORMAD2-AS1 interact with pivotal regulatory molecules including EZH2, AKT, and YAP1. EZH2, a histone methyltransferase, has been implicated in the progression of fibrosis, while YAP1, a core component of the Hippo signaling pathway, plays a critical role in liver regeneration and fibrogenesis [14]. Notably, circ_0057558 and lncRNA MALAT1 have been shown to promote hepatic lipid accumulation and metabolic dysfunction by sponging miR-206, a key regulator of lipid metabolism and insulin sensitivity [12,13]. The consistent upregulation of miR-206 in our MASLD cohort may therefore reflect compensatory regulatory feedback within ceRNA networks involving these or other uncharacterized transcript variants. Other studies demonstrate that transcript variants derived from lncRNAs such as LINC01234, LINC01138, and CYTOR can generate multiple isoforms with diverse ceRNA functions, influencing hepatic stellate cell activation, extracellular matrix remodeling, and the fibrotic response, central events in MASLD pathogenesis [14,15,57]. In other study, CYTOR has been shown to participate in YAP1-mediated pathways, enhancing fibrogenic gene expression, while alternative transcript isoforms of WDFY3-AS2 may exert protective effects by dampening pro-fibrotic signaling cascades through competitive miRNA binding [59]. Our canonical pathway analysis indicates strong associations between several ncRNAs (LINC00963, HCG18, ST7-AS1, RP11_25D31, CYTOR, and LINC01234) and hepatic fibrosis pathways. The notable upregulation of WDFY3-AS2 and LINC02767 suggests that these lncRNAs may contribute to hepatic inflammation and fibrotic remodeling, although further validation is required in larger populations [73].

Although our study offers new hypotheses on the transcriptomic landscape of MASLD in AAs and demonstrates the utility of whole-blood samples (Figure 6) for such investigations, it also has some limitations. First, transcriptomic profiling was performed using whole blood, which may not fully reflect hepatocyte- or hepatic stellate cell-specific regulatory processes occurring within the liver; thus, direct concordance between circulating and hepatic ncRNA expression cannot be assumed. Second, matched liver tissue was not available from the same individuals, precluding direct blood–liver cross-tissue correlation analyses to confirm whether dysregulated circulating miRNAs (e.g., miR-206, miR-224-5p, and miR-193a-5p) mirror hepatic expression patterns or target-gene regulation in situ. In addition, while pathway analyses and the prior literature support biologically plausible miRNA–target interactions, including miR-206 involvement in FGF21- and AMPK-associated metabolic signaling, this study did not include direct mechanistic validation using luciferase reporter assays or functional perturbation models. Future investigations should therefore incorporate paired blood–liver sampling, leverage publicly available MASLD cross-tissue transcriptomic datasets, and apply experimental validation approaches to strengthen causal inference and further justify whole blood as a surrogate tissue for hepatic regulatory profiling. Despite these limitations, the reproducibility across discovery and validation cohorts supports the robustness of the identified circulating ncRNA signature as a non-invasive biomarker framework for early-stage MASLD.

## 4. Materials and Methods

### 4.1. Study Participants and Blood Sample Collection

In this pilot study, the study participants consisted of eight individuals (control *n* = 4; MASLD *n* = 4), with equal numbers of males and females, who self-identified as AA and were born in the USA. All participants responded to an advertisement through Howard University and Georgetown University Community Newsletter via email and/or flyers and public announcements and were recruited with their informed consent. The protocol was approved by Georgetown-MedStar IRB (MODCR00002260). Participants with MASLD were recruited from the MedStar Georgetown Transplant Institute (Supplementary Table S2). We selected only those patients who were at early stages of development MASLD (confirmed hepatic steatosis and exhibiting one or more comorbid metabolic features, viz., type 2 diabetes, hypertension = SBP ≥ 140 mmHg/DBP ≥ 90 mmHg, dyslipidemia = LDL ≥ 100 mg/dL or triglycerides ≥ 150 mg/dL (ATP III criteria), or obesity). Individuals with severe fibrosis or cirrhosis were not included because of the small size of the participant groups and the wide spectrum of tissue features present during different stages of liver disease; we chose to limit subject inclusion to earlier stages of liver disease to increase the homogeneity of disease presentation in the different subjects. Individuals with severe fibrosis or cirrhosis were not included; we chose to limit subject inclusion to earlier stages of liver disease to increase the homogeneity of disease presentation in the different subjects (Table 1 and Table 2). Patients with other potential causes of liver disease, including viral, immunological, iron storage disease, Wilson disease, or alpha 1 antitrypsin deficiency, were excluded from the study. Participants with heavy alcohol use were also excluded from the study (alcohol intake > 21 drinks/week (males)/ > 14 (females)). Control participants were those who responded to the same flyers and advertisements described above but did not have MASLD and normal ALT/AST (<40 U/L). These individuals had negative HCV and HBV serology and had normal liver enzyme profiles. A questionnaire was provided to all participants to collect demographic and clinical information. Intergroup comparisons were assessed using Chi-square tests, and the corresponding *p*-values were calculated in Table 1 and Table 2.

### 4.2. RNA Extraction and miRNA Library Preparation

Whole blood was collected in a DNA/RNA Shield™ Blood Collection Tube (Manufacturer: Zymo Research, Irvine, CA, USA, Cat # R1150) during recruitment by experienced phlebotomists. Blood collection tubes were prefilled with 6 mL DNA/RNA Shield™ for direct collection of up to 3 mL whole human blood. DNA/RNA Shield lyses cells, inactivate nucleases and infectious agents (e.g., viruses and pathogens), and is ideal for safe sample storage and transport at ambient temperatures. RNA was extracted from DNA/RNA Shield tubes using the Quick-DNA/RNA™ Blood Tube Kit (Zymo Research, Irvine, CA, USA, Cat. # R1151) according to the manufacturer’s instructions. DNA contamination was removed using an Applied Biosystems Inc. (ABI) DNA-free kit (ThermoFisher, Foster City, CA, USA, Cat # AM 1906). RNA was quantified using a NanoDrop™ One spectrophotometer (Thermo Fisher Scientific, Wilmington, DE, USA). The ratio of absorbance at 260 and 280 nm was used to assess the purity of DNA and RNA. RNA integrity was assessed using an Agilent Bioanalyzer, and only samples with RNA Integrity Number (RIN) ≥ 7.0 were included for library preparation.

We used Zymo-Seq™ miRNA Library Kit (Zymo Research, Irvine, CA, USA Catalog Numbers: R3006, R3007) to generate small RNA libraries. To acquire miRNAs, present in total RNA or cell-free RNA extracted from biofluids, we followed the manufacturer’s instructions and protocol. Briefly, the protocol was with five major steps, which are adapter ligation and blocking, circularization and dimer removal, reverse transcription, index PCR, and library purification. At the time of index PCR, we pre-mixed forward and reverse primers with the following sequence:

Forward primer sequence:

5′-AATGATACGGCGACCACCGAGATCTACACNNNNNNNN(NN)ACAC TCTTTCCCTACACGACGCTCTTCCGATCT-3′;

Reverse primer sequence:

5′-CAAGCAGAAGACGGCATACGAGATNNNNNNNN(NN)GTGACTGGA GTTCCTTGGCACCCGAGAATTCCA-3′.

### 4.3. Sequencing and Data Analysis

The sequencing was performed by Zymo Research (Irvine, CA, USA). Data analysis was performed according to instructions mentioned in the Zymo-Seq™ miRNA Library Kit (Zymo Research, Irvine, CA, USA, Catalog Numbers: R3006, R3007). To read the Zymo-Seq™ miRNA libraries, we used bioinformatics tools (QIAGEN CLC Genomics Workbench 26.0.2 (Cambridge, MA, USA)) designed for Illumina’s TruSeq Small RNA libraries. Prior to sequence alignment, sequenced reads were processed with adapter trimming. Only high-quality sequencing reads corresponding to small RNA fragments of 18–30 nucleotides were retained for downstream analysis. Reads were required to meet a minimum base quality threshold of Q30 for ≥90% of bases to ensure high sequencing fidelity. For the trimming, we used sequence of TGGAATTCTCGGGTGCCAAGG. In the final analysis, data were extracted in the form of fold change and *p*-values. Differentially expressed miRNAs and other ncRNAs were identified using a fold-change threshold ≥ 1.5 and a false discovery rate (FDR) < 0.05, calculated using the Benjamini–Hochberg multiple testing correction method.

### 4.4. Ingenuity Pathway Analysis (IPA)

IPA was utilized to explore complex biofunctions within a biological system, identifying functional roles, molecular processes, and key networks associated with significantly differentially expressed genes in participants with MASLD. From the differential expression of miRNAs and other ncRNAs datasets described above, the identification of cellular processes and pathways by IPA (Qiagen, Cambridge, MA, USA) was performed according to the methods described in our earlier study [73,74,75]. Briefly, datasets comprising miRNA and other ncRNA identifiers and corresponding expression values (fold-change) from the sequencing data were imported into IPA. Differentially expressed identifiers (miRNAs and other ncRNAs) were mapped to related changes in biofunctions [74]. Only molecules meeting a fold-change threshold of ≥1.5 and a false discovery rate (FDR) < 0.05 were included in IPA-based biofunction, canonical pathway, and network analyses. The networks were generated algorithmically based on their connectivity. Using IPA, we identified the top network by amalgamating a large set of differentially expressed miRNAs and other ncRNAs with the goal of uncovering the most extensive array of relationships among the focus genes [76]. A score (P-score = −log10 (*p*-value)) according to the fit of the set of supplied genes and a list of biological functions stored in the Ingenuity Knowledge Base are generated [73] Networks were “named” on the most prevalent functional group(s) present. Canonical pathway analysis identified function-specific genes that were significantly present within the networks.

### 4.5. qRT-PCR Validation Study

In the qRT-PCR validation study, 30 participants were included (14 from the MASLD group, 16 from the control group). We used Applied Biosystem^®^ pre-configured TaqMan array card (96-well array card format) to examine the expression of genes of interest, viz., 18s (manufacturing control), GAPDH (internal control), miR-206, miR-1343-5p, miR-224-5p, miR1299, miR-193a-5p, miR185-3p, and miR-218-5p, as identified based on the global expression data and list of significant miRNA expressed in all samples. Six samples per plate were analyzed by High-hroughput FAST Real-Time PCR (Applied Biosystems, Foster City, CA, USA). qRT-PCR was carried out on a QuantStudio 7 Pro PCR system (Applied Biosystems, Foster City, CA, USA). The qRT-PCR mixture contained 5 μL of TaqMan™ Fast Advanced Master mix (Part # 4444557, Applied Biosystems, Foster City, California, USA) and 5 μL of cDNA diluted sample (as recommended by Taqman advanced miRNA assays protocol, Publication Number 100027897). All qRT-PCR reactions were performed in triplicate. Relative miRNA expression levels were calculated using the 2^−ΔΔCt^ method, with 18S and GAPDH serving as endogenous controls for normalization. Group comparisons between MASLD and control participants were performed using unpaired two-tailed Student’s *t*-tests. A *p*-value < 0.05 was considered statistically significant. Data are presented as the mean ± SEM from triplicate technical replicates. We used GAPDH as a reference gene because total RNA (including small RNAs) was isolated using a single-column extraction protocol, and cDNA synthesis was performed using the TaqMan™ Advanced miRNA system, which enables poly(A) tailing and universal reverse transcription across RNA classes. Under these conditions, GAPDH has been shown to exhibit stable expression across whole-blood samples and disease states and has been previously validated as an appropriate normalizer in circulating miRNA studies using TaqMan Advanced assays. Although small nuclear RNAs such as U6 are commonly used as internal controls in tissue-based or enriched small RNA preparations, their expression can be variable in whole blood and circulating RNA contexts, particularly across inflammatory and metabolic conditions. Schwarzenbach et al. emphasized that no universal reference gene is suitable for all miRNA studies and recommended empirical validation of endogenous controls, especially in blood-based and circulating RNA analyses [77]. Similarly, Iorio et al. demonstrated that reference gene stability may vary substantially across pathological conditions, reinforcing the need for context-specific normalization strategies [78]. Consistent with these recommendations, GAPDH and 18S rRNA were selected in this study based on their reported expression stability in total RNA-based workflows and their compatibility with poly(A)-tailing and universal reverse transcription approaches used in TaqMan™ Advanced miRNA assays [78,79]. To further support their suitability in the present cohort, expression stability of GAPDH and 18S rRNA was evaluated across all validation samples. Ct values for both endogenous controls demonstrated low inter-sample variability and no statistically significant differences between MASLD and control groups, supporting their use as stable normalization controls for circulating miRNA analysis in this study.

### 4.6. Statistical Analysis

Clinical and demographic variables were summarized as the mean ± standard deviation (SD) or mean ± SEM, as appropriate. Between-group comparisons for continuous variables were performed using unpaired two-tailed Student’s *t*-tests, while categorical variables were compared using Chi-square tests. Statistical analyses were conducted using GraphPad Prism (version 10). A *p*-value < 0.05 was considered statistically significant.

## 5. Conclusions

Our study is among the first to generate hypotheses on the significance of miRNA and ncRNA expression patterns in MASLD among AAs. The pathway analyses reveal correlations between these ncRNA patterns and the transcriptomic patterns of coding genes, particularly in pathways involving TGFB1 and E2F signaling. The findings also suggest that miR-206 upregulation may represent a protective response to insulin resistance. miR-206, miR-370-3p, and miR-193a-5p downregulation could contribute to MASLD pathogenesis via impaired lipid metabolism and fibrosis promotion. Other ncRNAs such as LINC00963, SNHG7, and CYTOR are implicated in hepatic fibrosis signaling. These findings highlight promising candidates for future biomarker investigation and therapeutic targeting. Further large-scale studies, including longitudinal transcriptomic profiling, will be essential to elucidate the role of ncRNAs in MASLD progression and ethnic disparities.

## Figures and Tables

**Table 1:** Participants characteristics.

*Variable*	Discovery Cohort			Validation Cohort		
	Control (*n* = 4)	MASLD (*n* = 4)	*p*-Value	Control (*n* = 16)	MASLD (*n* = 14)	*p*-Value
*Age (years)*	61 ± 4.83	53 ± 5.94	0.08	53 ± 12.97	46 ± 8.36	0.08
*Male/female*	2/2	2/2	-	6/10	6/8	-
*BMI (kg/m^2^)*	25.22 ± 1.53	27.8 ± 4.25	0.29	30.61 ± 8.38	30.26 ± 7.81	0.90
*Hba1c (%)*	5.26 ± 0.37	5.65 ± 0.07	0.27	5.35 ± 0.57	6.58 ± 1.56	0.01
*LDL (optimal range < * 100 mg/dL) *	-	137.34 ± 26.65	-	-	102.5 ± 49.17	-
HDL (optimal range 40–70 mg/dL) *	-	50.34 ± 18.82	-	-	48.93 ± 17.94	-
*Triglyceride (optimal range < * *150 mg/dL) * *	-	124 ± 85.08	-	-	138.64 ± 75.11	-

* LDL, HDL, and triglyceride data were not collected for control participants because these parameters were within normal reference ranges according to their medical records.

**Table 2:** Fibrosis/steatosis stages of MASLD patients.

Variable	Category	Discovery Cohort (Total *n* = 4)	Validation Cohort (Total *n* = 14)	*p*-Value
Fibrosis stage *	F0	1	3	0.108
	F0–F1	1	4	
	F2	0	5	
	F2–F3	2	0	
	F3	0	1	
	F4	0	1	
Steatosis stage **	S0	0	5	0.097
	S1	1	0	
	S1–S2	0	3	
	S2	0	2	
	S2-S3	0	1	
	S3	3	3	

* F0—normal, F1—mild fibrosis, F3—moderate fibrosis, and F4—severe fibrosis. FibroScan and steatosis data were not collected for control participants because these parameters were within normal reference ranges according to their medical records. ** S0—steatosis less than 11% (normal), S1—steatosis 11% to 33%, S2—steatosis 34% to 66%, and S3—steatosis greater than 67%.

**Table 3:** Differentially expressed miRNAs in MASLD subjects compared to controls.

miRNA	Fold Change	*p*-Value	Biological Functions
*hsa-miR-218-5p*	−5.82	0.0003	Regulates placental development, airway inflammation, and hepatic lipogenesis; targets TGFβ2, SMAD2, TLR4, and Elovl5 [16].
*hsa-miR-370-3p*	−3.88	0.0005	Regulates VSMC phenotype, glioblastoma suppression, and sinus node dysfunction in heart failure [17].
*hsa-miR-4731-5p*	−3.57	0.0125	Tumor suppressor in glioblastoma, melanoma, and NSCLC; impacts viability, EMT, and apoptosis [18].
*hsa-miR-1343-5p*	−3.48	0.0020	Reduces TGF-β signaling and fibrosis via exosomal delivery; therapeutic potential in lung disease [19].
*hsa-miR-224-5p*	−2.88	0.0001	Promotes EMT in hepatocellular carcinoma, regulates autophagy in breast cancer, and modulates cardiovascular inflammation [20].
*hsa-miR-193a-5p*	−2.80	0.0031	Tumor suppressor; inhibits proliferation and metastasis in ovarian and prostate cancers [21].
*hsa-miR-1299*	−2.78	0.0055	Tumor suppressor; inhibits NEK2 in prostate cancer and also regulates RHOT1 and PDL1 in other cancers [22].
*hsa-miR-4707-3p*	−2.69	0.0021	Modulates cell fate in human neocortex development [23].
*hsa-miR-133a-3p*	−2.67	0.0247	Tumor suppressor in colorectal cancer; inhibits angiogenesis [24].
*hsa-miR-365a-3p*	−2.59	0.0236	Promotes lung cancer via PI3K/AKT; affects osteogenesis by targeting RUNX2 [25].
*hsa-miR-4664-5p*	−2.59	0.0223	Detected in breast cancer; potential cancer biomarker [26].
*hsa-miR-539-5p*	−2.51	0.0039	Inhibits pancreatic cancer proliferation; regulates Tregs in leukemia [27].
*hsa-miR-369-5p*	−2.37	0.0175	Inhibits hepatocellular carcinoma by targeting HOXA13 [28].
*hsa-miR-150-3p*	−2.12	0.0275	Antitumor in lung cancer; enhances neuronal proliferation [29].
*hsa-miR-1185-1-3p*	−2.05	0.0267	Biomarker for weight loss response; associated with lung cancer [30].
*hsa-miR-3940-3p*	−2.01	0.0026	Promotes granulosa cell proliferation; linked to insulin resistance in pregnancy [31].
*hsa-miR-369-3p*	−1.90	0.0373	Anti-inflammatory; inhibits preadipocyte proliferation and differentiation [32].
*hsa-miR-452-5p*	−1.89	0.0297	Regulates fibrosis and promotes cancer progression [33].
*hsa-miR-323b-3p*	−1.86	0.0363	Upregulated in Huntington’s disease; involved in neurodegeneration [34].
*hsa-miR-433-3p*	−1.82	0.0236	Suppresses glioma growth; enhances chemotherapy sensitivity [35].
*hsa-miR-379-5p*	−1.81	0.0209	Plays a role in regulating cellular processes, particularly in cancer development and progression [36].
*hsa-miR-409-5p*	−1.71	0.0480	Promotes tumor growth, EMT, and bone metastasis in prostate cancer [37].
*hsa-miR-487b-3p*	−1.69	0.0112	Negative regulator of skeletal myogenesis; suppresses C2C12 myoblast proliferation [38].
*hsa-miR-154-5p*	−1.65	0.0451	Triggers cardiac oxidative stress and inflammation; tumor suppressor in glioblastoma [39].
*hsa-miR-3195*	1.60	0.0125	Suppresses osteosarcoma progression by targeting SOX4; linked to prostate cancer [40].
*hsa-miR-6758-5p*	1.65	0.0165	Specific function remains unknown.
*hsa-miR-4479*	1.7	0.0198	Potential biomarker in cancer; roles in immunosuppression and metastasis [41].
*hsa-miR-196a-5p*	1.7	0.0437	Oncogene; promotes invasion, metastasis, and proliferation in many cancers [42].
*hsa-miR-4762-5p*	2.0	0.0034	Detected in breast cancer tissues; role in tumorigenesis is under study [43].
*hsa-miR-129-5p*	2.35	0.0147	Tumor suppressor; inhibits proliferation in hepatocellular carcinoma [44].
*hsa-miR-206*	2.56	0.0353	Involved in cancers, neurodegenerative, and cardiovascular diseases; tumor suppressor [45].
*hsa-miR-4645-5p*	3.02	0.0309	Facilitates diabetic wound healing by restoring keratinocyte autophagy [46].
*hsa-miR-561-3p*	3.80	0.0122	Modulates CX3CL1 signaling in hepatocellular carcinoma; suppresses metastasis [47].
*hsa-miR-4669*	3.85	<0.0001	Enhances tumor aggressiveness creates immunosuppressive environment in liver cancer [48].
*hsa-miR-5698*	5.29	<0.0001	Identified as breast cancer biomarker; functions not well characterized [49].

The above list of miRNAs includes those that are differentially expressed in the MASLD group (*n* = 4) compared to the control group (*n* = 4), with a fold-change cutoff of ±1.5 (or at least 1.5-fold) and a *p*-value < 0.05.

**Table 4:** Other differentially expressed ncRNAs compared to controls.

Other ncRNA	Fold Change	*p*-Value	Biological Functions
*Homo_sapiens_tRNA-Leu-AAG-1*	−8.03	0.043	Encodes a tRNA specific for leucine with the AAG anticodon, essential for protein synthesis.
*ENSG00000282021*	−6.29	0.004	Specific function remains unknown.
*ENSG00000285756*	−5.95	0.006	Specific function remains unknown.
*DLX6-AS1*	−5.76	0.009	Long non-coding RNA implicated in promoting tumor cell proliferation, migration, invasion, and epithelial–mesenchymal transition in various cancers [50].
*FMNL1-DT*	−5.44	0.034	Specific function remains unknown.
*APOBEC3B-AS1*	−5.42	0.003	Specific function remains unknown.
*RN7SL426P*	−5.23	0.012	Specific function remains unknown.
*ENSG00000254639*	−5.23	0.020	Specific function remains unknown.
*RSF1-IT1*	−5.20	0.020	Specific function remains unknown.
*ENSG00000273064*	−5.07	0.036	Specific function remains unknown.
*PRDM16-DT*	−5.03	0.031	Long non-coding RNA involved in regulating astrocyte function and implicated in colorectal cancer metastasis and drug resistance [51].
*RNU6-70P*	−5.02	0.025	Specific function remains unknown.
*Homo_sapiens_tRNA-Gly-GCC-5*	−4.41	0.005	Encodes a tRNA specific for glycine with the GCC anticodon, essential for protein synthesis.
*U8*	−3.75	0.019	Specific function remains unknown.
*NFE4*	−3.11	0.014	Transcription factor involved in regulating fetal γ-globin gene expression; acetylation of NFE4 prevents its ubiquitination and modulates its interaction with histone deacetylase HDAC1, influencing gene activation [52].
*Homo_sapiens_tRNA-Met-CAT-6*	−1.95	0.037	Encodes transfer RNA for methionine with anticodon CAT, essential for initiating protein synthesis.
*Homo_sapiens_tRNA-Asp-GTC-2*	−1.86	0.002	Encodes transfer RNA for aspartic acid with anticodon GTC, facilitating incorporation of aspartic acid during protein synthesis.
*SNORD99*	1.69	0.007	Small nucleolar RNA involved in 2′-O-methylation of ribosomal RNA; overexpression promotes endometrial cancer development by inhibiting GSDMD-mediated pyroptosis [53].
*SNORD96A*	1.71	0.005	Small nucleolar RNA implicated in ribosomal RNA modification; elevated levels in plasma serve as a non-invasive diagnostic biomarker for clear cell renal cell carcinoma (ccRCC) [54].
*SNORD48*	1.71	0.030	Small nucleolar RNA involved in post-transcriptional modification of other small nuclear RNAs; associated with prostate and hematologic cancers [55].
*ENSG00000280434*	1.97	0.004	Specific function remains unknown.
*SNHG29*	2.40	0.000	Long non-coding RNA that regulates cell senescence via p53/p21 signaling and promotes glioblastoma progression through the miR-223-3p/CTNND1 axis [56].
*LINC01138*	2.74	0.012	Long intergenic non-coding RNA that acts as an oncogenic driver by interacting with PRMT5, enhancing its stability, and promoting tumorigenicity in hepatocellular carcinoma [57].
*ENSG00000253374*	3.86	0.033	Specific function remains unknown.
*RN7SL33P*	4.58	0.021	Specific function remains unknown.
*LINC00461*	4.96	0.000	Long non-coding RNA important for glioma progression, affecting cell proliferation, migration, and invasion via MAPK/ERK and PI3K/AKT signaling pathways [58].
*ENSG00000286834*	5.09	0.012	Specific function remains unknown.
*WDFY3-AS2*	5.28	0.022	Long non-coding RNA that acts as a tumor suppressor by inhibiting cell proliferation and metastasis through the Wnt/β-catenin signaling pathway in oral squamous cell carcinoma [59].

The above list of ncRNAs includes those that are differentially expressed in the MASLD group (*n* = 4) compared to the control group (*n* = 4), with a fold-change cutoff of ±1.5 (or at least 1.5-fold) and a *p*-value < 0.05.

**Table 5:** MicroRNAs differentially expressed in all MASLD subjects.

miRNA ID	Fold Change	*p*-Value	Role in MASLD
*miR-206*	2.22 ± 0.19	0.0353	miR-206 regulates lipid metabolism and fibrosis in MASLD by downregulating FGF21 and modulating the MAPK pathway.
*miR-1343-5p*	−3.98 ± 2.50	0.0020	miR-1343-5p contributes to MASLD by modulating the PI3K/Akt pathway, promoting hepatic lipid accumulation and inflammation.
*miR-224-5p*	−2.65 ± 0.52	0.0001	miR-224-5p exacerbates MASLD by activating the TGF-β/Smad pathway, promoting liver fibrosis and inflammation.
*miR-1299*	−3.59 ± 1.71	0.0055	miR-1299 plays a role in MASLD by inhibiting the Wnt/β-catenin pathway, thereby reducing hepatic fibrosis and lipid accumulation.
*miR-193a-5p*	−1.79 ± 0.26	0.0031	miR-193a-5p contributes to MASLD by deactivating the JNK/c-Jun pathway, which reduces inflammation and hepatic injury.
*miR-185-3p*	−2.59 ± 1.06	0.0038	miR-185-3p mitigates MASLD by inhibiting the NF-κB pathway, reducing inflammation and liver damage.
*miR-3960*	−1.64 ± 0.95	0.0270	miR-3960 contributes to MASLD by activating the SIRT1/AMPK pathway, promoting lipid metabolism and reducing hepatic steatosis.

## Data Availability

The data supporting the findings of this study are available from the corresponding author upon reasonable request.

## Supplementary Materials

The following supporting information can be downloaded at: https://www.mdpi.com/article/10.3390/ijms27041666/s1.

## References

[1] Younossi Z.M., Stepanova M., Afendy M., Fang Y., Younossi Y., Mir H., Srishord M. (2019). Changes in the prevalence of the most common causes of chronic liver diseases in the United States from 1988 to 2018. Clin. Gastroenterol. Hepatol..

[2] Chalasani N., Younossi Z., Lavine J.E., Diehl A.M., Brunt E.M., Cusi K., Rinella M., Harrison S.A., Brunt E.M., Sanyal A.J. (2021). The diagnosis and management of nonalcoholic fatty liver disease: Practice guidance from the American Association for the Study of Liver Diseases. Hepatology.

[3] Diehl A.M., Day C. (2020). Cause, pathogenesis, and treatment of nonalcoholic steatohepatitis. N. Engl. J. Med..

[4] Iruzubieta P., Jimenez-Gonzalez C., Cabezas J., Crespo J. (2025). From NAFLD to MASLD: Transforming steatotic liver disease diagnosis and management. Metab. Target Organ Damage.

[5] Buzzetti E., Pinzani M., Tsochatzis E.A. (2016). The multiple-hit pathogenesis of non-alcoholic fatty liver disease (NAFLD). Metab. Clin. Exp..

[6] Sunny N.E., Parks E.J., Browning J.D., Burgess S.C. (2011). Excessive hepatic mitochondrial TCA cycle and gluconeogenesis in humans with nonalcoholic fatty liver disease. Cell Metab..

[7] Rich N.E., Oji S., Mufti A.R., Browning J.D., Parikh N.D., Singal A.G. (2022). Racial and ethnic disparities in nonalcoholic fatty liver disease prevalence, severity, and outcomes in the United States: A systematic review and meta-analysis. Clin. Gastroenterol. Hepatol..

[8] Mondal T., Smith C.I., Loffredo C.A., Quartey R., Moses G., Howell C.D., Korba B., Kwabi-Addo B., Nunlee-Bland G., RRucker L. (2023). Transcriptomics of MASLD Pathobiology in African American Patients in the Washington DC Area. Int. J. Mol. Sci..

[9] Mondal T., Loffredo C.A., Simhadri J., Nunlee-Bland G., Korba B., Johnson J., Cotin S., Moses G., Quartey R., Howell C.D. (2023). Insights on the pathogenesis of type 2 diabetes as revealed by signature genomic classifiers in an African American population in the Washington, DC area. Diabetes/Metab. Res. Rev..

[10] Bartel D.P. (2004). MicroRNAs: Genomics, biogenesis, mechanism, and function. Cell.

[11] O’Brien J., Hayder H., Zayed Y., Peng C. (2018). Overview of MicroRNA Biogenesis, Mechanisms of Actions, and Circulation. Front. Endocrinol..

[12] Chen X., Tan Q.Q., Tan X.R., Li S.J., Zhang X.X. (2021). Circ_0057558 promotes nonalcoholic fatty liver disease by regulating ROCK1/AMPK signaling through targeting miR-206. Cell Death Dis..

[13] Xiang J., Deng Y.Y., Liu H.X., Pu Y. (2022). LncRNA MALAT1 Promotes PPARα/CD36-Mediated Hepatic Lipogenesis in Nonalcoholic Fatty Liver Disease by Modulating miR-206/ARNT Axis. Front. Bioeng. Biotechnol..

[14] Yuan Z., Meng J., Shen X., Wang M., Yu Y., Shi L., Li Y.L., Hassan H.M., Li H., He Z.X. (2024). Formononetin Mitigates Liver Fibrosis via Promoting Hepatic Stellate Cell Senescence and Inhibiting EZH2/YAP Axis. J. Agric. Food Chem..

[15] Wang Z., Yang X., Gui S., Yang F., Cao Z., Cheng R., Xia X., Li C. (2021). The Roles and Mechanisms of lncRNAs in Liver Fibrosis. Front. Pharmacol..

[16] Wu G., Zhang Y., Liang B., Yin L., Gao M., Zhang H., Xu Y., Han X., Qi Y., Liu F. (2024). miR-218-5p promotes hepatic lipogenesis through targeting Elovl5 in non-alcoholic fatty liver disease. Biochem. Pharmacol..

[17] Yanni J., D’Souza A., Wang Y., Li N., Hansen B.J., Zakharkin S.O., Smith M., Hayward C., Whitson B.A., Mohler P.J. (2020). Silencing miR-370-3p rescues funny current and sinus node function in heart failure. Sci. Rep..

[18] Allahverdi A., Arefian E., Soleimani M., Ai J., Nahanmoghaddam N., Yousefi-Ahmadipour A., Ebrahimi-Barough S. (2020). MicroRNA-4731-5p delivered by AD-mesenchymal stem cells induces cell cycle arrest and apoptosis in glioblastoma. J. Cell. Physiol..

[19] Stolzenburg L.R., Harris A. (2018). Microvesicle-mediated delivery of miR-1343: Impact on markers of fibrosis. Cell Tissue Res..

[20] Gozuacik D., Akkoc Y., Ozturk D.G., Kocak M. (2017). Autophagy-Regulating microRNAs and Cancer. Front. Oncol..

[21] Tang W., Rao Y., Pi L., Li J. (2025). A review on the role of MiR-193a-5p in oncogenesis and tumor progression. Front. Oncol..

[22] Zhang F.B., Du Y., Tian Y., Ji Z.G., Yang P.Q. (2019). MiR-1299 functions as a tumor suppressor to inhibit the proliferation and metastasis of prostate cancer by targeting NEK2. Eur. Rev. Med. Pharmacol. Sci..

[23] Lafferty M.J., Aygün N., Patel N.K., Krupa O., Liang D., Wolter J.M., Geschwind D.H., de la Torre-Ubieta L., Stein J.L. (2023). MicroRNA-eQTLs in the developing human neocortex link miR-4707-3p expression to brain size. eLife.

[24] Yin Y., Du L., Li X., Zhang X., Gao Y. (2019). miR-133a-3p suppresses cell proliferation, migration, and invasion and promotes apoptosis in esophageal squamous cell carcinoma. J. Cell. Physiol..

[25] Cheng F., Yang M.M., Yang R.H. (2019). MiRNA-365a-3p promotes the progression of osteoporosis by inhibiting osteogenic differentiation via targeting RUNX2. Eur. Rev. Med. Pharmacol. Sci..

[26] Hu Y., Dingerdissen H., Gupta S., Kahsay R., Shanker V., Wan Q., Yan C., Mazumder R. (2018). Identification of key differentially expressed MicroRNAs in cancer patients through pan-cancer analysis. Comput. Biol. Med..

[27] Dai Q., Shi R., Zhang G., Wang Y., Ye L., Peng L., Guo S., He J., Yang H., Jiang Y. (2024). miR-539-5p targets BMP2 to regulate Treg activation in B-cell acute lymphoblastic leukemia through TGF-β/Smads/MAPK. Exp. Biol. Med..

[28] Qian X., Wang Y., Hu W., Xu X., Gao L., Meng Y., Yan J. (2022). MiR-369-5p inhibits the proliferation and migration of hepatocellular carcinoma cells by down-regulating HOXA13 expression. Tissue Cell.

[29] Zhang N., Wei X., Xu L. (2013). miR-150 promotes the proliferation of lung cancer cells by targeting P53. FEBS Lett..

[30] Ma J., Mannoor K., Gao L., Tan A., Guarnera M.A., Zhan M., Shetty A., Stass S.A., Xing L., Jiang F. (2014). Characterization of microRNA transcriptome in lung cancer by next-generation deep sequencing. Mol. Oncol..

[31] Alvarado-Flores F., Kaneko-Tarui T., Beyer W., Katz J., Chu T., Catalano P., Sadovsky Y., Hivert M.-F., O’tIerney-Ginn P. (2021). Placental miR-3940-3p Is Associated with Maternal Insulin Resistance in Late Pregnancy. J. Clin. Endocrinol. Metab..

[32] Xue S., Liu K., Zhao L., Zhou L., Gao X., Liu L., Liu N., He J. (2023). The role of miR-369-3p in proliferation and differentiation of preadipocytes in Aohan fine-wool sheep. Arch. Anim. Breed..

[33] Mushtaq I., Hsieh T.H., Chen Y.C., Kao Y.H., Chen Y.J. (2024). MicroRNA-452-5p regulates fibrogenesis via targeting TGF-β/SMAD4 axis in SCN5A-knockdown human cardiac fibroblasts. iScience.

[34] Ferraldeschi M., Romano S., Giglio S., Romano C., Morena E., Mechelli R., Annibali V., Ubaldi M., Buscarinu M.C., Umeton R. (2021). Circulating hsa-miR-323b-3p in Huntington’s Disease: A Pilot Study. Front. Neurol..

[35] Sun S., Wang X., Xu X., Di H., Du J., Xu B., Wang Q., Wang J. (2017). MiR-433-3p suppresses cell growth and enhances chemosensitivity by targeting CREB in human glioma. Oncotarget.

[36] Meng L., Du Y., Deng B., Duan Y. (2023). miR-379-5p regulates the proliferation, cell cycle, and cisplatin resistance of oral squamous cell carcinoma cells by targeting ROR1. Am. J. Transl. Res..

[37] Josson S., Gururajan M., Hu P., Shao C., Chu G.C.-Y., Zhau H.E., Liu C., Lao K., Lu C.-L., Lu Y.-T. (2014). miR-409-3p/-5p promotes tumorigenesis, epithelial-to-mesenchymal transition, and bone metastasis of human prostate cancer. Clin. Cancer Res..

[38] Wang J., Tan J., Qi Q., Yang L., Wang Y., Zhang C., Hu L., Chen H., Fang X. (2018). miR-487b-3p Suppresses the Proliferation and Differentiation of Myoblasts by Targeting IRS1 in Skeletal Muscle Myogenesis. Int. J. Biol. Sci..

[39] Wang Q., Yu X., Dou L., Huang X., Zhu K., Guo J., Yan M., Wang S., Man Y., Tang W. (2019). miR-154-5p Functions as an Important Regulator of Angiotensin II-Mediated Heart Remodeling. Oxid. Med. Cell Longev..

[40] Liang J., Bao D., Ye Z., Cao B., Jin G., Lu Z., Chen J. (2023). miR-3195 suppresses the malignant progression of osteosarcoma cells via targeting SOX4. J. Orthop. Surg. Res..

[41] Wang S., Song X., Wang K., Zheng B., Lin Q., Yu M., Xie L., Chen L., Song X. (2022). Plasma exosomal miR-320d, miR-4479, and miR-6763-5p as diagnostic biomarkers in epithelial ovarian cancer. Front. Oncol..

[42] Xin H., Wang C., Liu Z. (2019). miR-196a-5p promotes metastasis of colorectal cancer via targeting IκBα. BMC Cancer.

[43] Jia F., Zhang L., Jiang Z., Tan G., Wang Z. (2023). FZD1/KLF10-hsa-miR-4762-5p/miR-224-3p-circular RNAs axis as prognostic biomarkers and therapeutic targets for glioblastoma: A comprehensive report. BMC Med. Genom..

[44] Li Z., Lu J., Zeng G., Pang J., Zheng X., Feng J., Zhang J. (2019). MiR-129-5p inhibits liver cancer growth by targeting calcium calmodulin-dependent protein kinase IV (CAMK4). Cell Death Dis..

[45] Khalilian S., Hosseini Imani S.Z., Ghafouri-Fard S. (2022). Emerging roles and mechanisms of miR-206 in human disorders: A comprehensive review. Cancer Cell Int..

[46] Shi Y., Wang S., Liu D., Wang Z., Zhu Y., Li J., Xu K., Li F., Wen H., Yang R. (2024). Exosomal miR-4645-5p from hypoxic bone marrow mesenchymal stem cells facilitates diabetic wound healing by restoring keratinocyte autophagy. Burn. Trauma.

[47] Chen E.B., Zhou Z.J., Xiao K., Zhu G.Q., Yang Y., Wang B., Zhou S.L., Chen Q., Yin D., Wang Z. (2019). The miR-561-5p/CX_3_CL1 Signaling Axis Regulates Pulmonary Metastasis in Hepatocellular Carcinoma Involving CX_3_CR1^+^ Natural Killer Cells Infiltration. Theranostics.

[48] Nakano T., Chen C.L., Chen I.H., Tseng H.P., Chiang K.C., Lai C.Y., Hsu L.W., Goto S., Lin C.C., Cheng Y.F. (2023). Overexpression of miR-4669 Enhances Tumor Aggressiveness and Generates an Immunosuppressive Tumor Microenvironment in Hepatocellular Carcinoma: Its Clinical Value as a Predictive Biomarker. Int. J. Mol. Sci..

[49] Sathipati S.Y., Tsai M.J., Aimalla N., Moat L., Shukla S.K., Allaire P., Hebbring S., Beheshti A., Sharma R., Ho S.-Y. (2024). An evolutionary learning-based method for identifying a circulating miRNA signature for breast cancer diagnosis prediction. NAR Genom. Bioinform..

[50] Ghafouri-Fard S., Najafi S., Hussen B.M., Ganjo A.R., Taheri M., Samadian M. (2022). DLX6-AS1: A Long Non-coding RNA With Oncogenic Features. Front. Cell Dev. Biol..

[51] Schröder S., Fuchs U., Gisa V., Pena T., Krüger D.M., Hempel N., Burkhardt S., Salinas G., Schütz A.L., Delalle I. (2024). PRDM16-DT is a novel lncRNA that regulates astrocyte function in Alzheimer’s disease. Acta Neuropathol..

[52] Zhao Q., Cumming H., Cerruti L., Cunningham J.M., Jane S.M. (2004). Site-specific acetylation of the fetal globin activator NF-E4 prevents its ubiquitination and regulates its interaction with the histone deacetylase, HDAC1. J. Biol. Chem..

[53] Xian J.Y., Wu W., Chen X., Bao H.J., Zhang S., Sheng X.J., Chen S. (2024). SNORD99 promotes endometrial cancer development by inhibiting GSDMD-mediated pyroptosis through 2′-O-methylation modification. J. Cell. Mol. Med..

[54] Shang X., Song X., Wang K., Yu M., Ding S., Dong X., Xie L., Song X. (2021). SNORD63 and SNORD96A as the non-invasive diagnostic biomarkers for clear cell renal cell carcinoma. Cancer Cell Int..

[55] Liang J., Wen J., Huang Z., Chen X.P., Zhang B.X., Chu L. (2019). Small Nucleolar RNAs: Insight Into Their Function in Cancer. Front. Oncol..

[56] Jiang J., Hu H., Chen Q., Zhang Y., Chen W., Huang Q., Chen X., Li J., Zhong M. (2021). Long non-coding RNA SNHG29 regulates cell senescence via p53/p21 signaling in spontaneous preterm birth. Placenta.

[57] Li Z., Zhang J., Liu X., Li S., Wang Q., Di Chen Hu Z., Yu T., Ding J., Li J., Yao M. (2018). The LINC01138 drives malignancies via activating arginine methyltransferase 5 in hepatocellular carcinoma. Nat. Commun..

[58] Yang Y., Ren M., Song C., Li D., Soomro S.H., Xiong Y., Zhang H., Fu H. (2017). LINC00461, a long non-coding RNA, is important for the proliferation and migration of glioma cells. Oncotarget.

[59] Lin X., Ding J.M., Zheng X.Z., Chen J.G. (2023). Immunity-related long noncoding RNA WDFY3-AS2 inhibited cell proliferation and metastasis through Wnt/β-catenin signaling in oral squamous cell carcinoma. Arch. Oral. Biol..

[60] Mohammed A., Shaker O.G., Khalil M.A.F., Abu-El-Azayem A.K., Samy A., Fathy S.A., AbdElguaad M.M.K., Mahmoud F.A.M., Erfan R. (2024). Circulating miR-206, miR-181b, and miR-21 as promising biomarkers in hypothyroidism and their relationship to related hyperlipidemia and hepatic steatosis. Front. Mol. Biosci..

[61] Cheung O., Puri P., Eicken C., Contos M.J., Mirshahi F., Maher J.W., Kellum J.M., Min H., Luketic V.A., Sanyal A.J. (2008). Nonalcoholic steatohepatitis is associated with altered hepatic MicroRNA expression. Hepatology.

[62] Zhou J., Wang H., Sun Q., Liu X., Wu Z., Wang X., Fang W., Ma Z. (2021). miR-224-5p-enriched exosomes promote tumorigenesis by directly targeting androgen receptor in non-small cell lung cancer. Mol. Ther. Nucleic Acids.

[63] Mehta R., Otgonsuren M., Younoszai Z., Allawi H., Raybuck B., Younossi Z. (2016). Circulating miRNA in patients with non-alcoholic fatty liver disease and coronary artery disease. BMJ Open Gastroenterol..

[64] Saini A., Rutledge B., Damughatla A.R., Rasheed M., Naylor P., Mutchnick M. (2024). Manifestation and Progression of Metabolic Dysfunction-Associated Steatotic Liver Disease in a Predominately African American Population at a Multi-Specialty Healthcare Organization. Healthcare.

[65] Li Y.J., Baumert B.O., Stratakis N., Goodrich J.A., Wu H.T., He J.X., Zhao Y.Q., Aung M.T., Wang H.X., Eckel S.P. (2024). Circulating microRNA expression and nonalcoholic fatty liver disease in adolescents with severe obesity. World J. Gastroenterol..

[66] Johnson K., Leary P.J., Govaere O., Barter M.J., Charlton S.H., Cockell S.J., Tiniakos D., Zatorska M., Bedossa P., Brosnan M.J. (2021). Increased serum miR-193a-5p during non-alcoholic fatty liver disease progression: Diagnostic and mechanistic relevance. JHEP Rep..

[67] Zhang X., Mens M.M.J., Abozaid Y.J., Bos D., Darwish Murad S., de Knegt R.J., Ikram M.A., Pan Q., Ghanbari M. (2021). Circulatory microRNAs as potential biomarkers for fatty liver disease: The Rotterdam study. Aliment. Pharmacol. Ther..

[68] Hochberg J.T., Sohal A., Handa P., Maliken B.D., Kim T.K., Wang K., Gochanour E., Li Y., Rose J.B., Nelson J.E. (2023). Serum miRNA profiles are altered in patients with primary sclerosing cholangitis receiving high-dose ursodeoxycholic acid. JHEP Rep. Innov. Hepatol..

[69] Behrooz M., Hajjarzadeh S., Kahroba H., Ostadrahimi A., Bastami M. (2023). Expression pattern of miR-193a, miR122, miR155, miR-15a, and miR146a in peripheral blood mononuclear cells of children with obesity and their relation to some metabolic and inflammatory biomarkers. BMC Pediatr..

[70] Zhang X., Zhang D., Bu X., Zhang X., Cui L. (2022). Identification of a novel miRNA-based recurrence and prognosis prediction biomarker for hepatocellular carcinoma. BMC Bioinform..

[71] Li H., Liu T., Yang Y., Cho W.C., Flynn R.J., Harandi M.F., Song H., Luo X., Zheng Y. (2022). Interplays of liver fibrosis-associated microRNAs: Molecular mechanisms and implications in diagnosis and therapy. Genes. Dis..

[72] Wang S., Chen Y., Lei G., Ma X., An L., Wang H., Song Z., Lin L., He Q., Xu R. (2024). Serum Exosome-Derived microRNA-193a-5p and miR-381-3p Regulate Adenosine 5′-Monophosphate-Activated Protein Kinase/Transforming Growth Factor Beta/Smad2/3 Signaling Pathway and Promote Fibrogenesis. Clin. Transl. Gastroenterol..

[73] Sur T.K., Mondal T., Noreen Z., Johnson J., Nunlee-Bland G., Loffredo C.A., Korba B.E., Chandra V., Jana S.S., Kwabi-Addo B. (2025). Developing Non-Invasive Molecular Markers for Early Risk Assessment of Alzheimer’s Disease. Biomark. Neuropsychiatry.

[74] Mondal T., Noreen Z., Loffredo C.A., Johnson J., Bhatti A., Nunlee-Bland G., Quartey R., Howell C.D., Moses G., Nnanabu T. (2024). Transcriptomic Analysis of Alzheimer’s Disease Pathways in a Pakistani Population. J. Alzheimers Dis. Rep..

[75] Mondal T., Johnson J., Sur T.K., Loffredo C.A., Cotin S.T., Sahota J., Korba B.E., Nunlee-Blnad G., Ghosh S. (2025). Metabolic Dysfunction and Alzheimer’s Disease Risks in African Americans. Alzheimer’s Dement..

[76] Liu C.H., Ampuero J., Gil-Gómez A., Montero-Vallejo R., Rojas A., Muñoz-Hernández R., Gallego-Durán R., Romero-Gómez M. (2021). miRNAs in patients with non-alcoholic fatty liver disease: A systematic review and meta-analysis. J. Hepatol..

[77] Schwarzenbach H., da Silva A.M., Calin G., Pantel K. (2015). Data normalization strategies for microRNA quantification. Clin. Chem..

[78] Iorio M.V., Visone R., Di Leva G., Donati V., Petrocca F., Casalini P., Taccioli C., Volinia S., Liu C.G., Alder H. (2007). MicroRNA signatures in human ovarian cancer. Cancer Res..

[79] Grenda A., Krawczyk P., Błach J., Chmielewska I., Kubiatowski T., Kieszko S., Wojas-Krawczyk K., Kucharczyk T., Jarosz B., Paśnik I. (2021). Tissue microRNA expression as a predictor of response to immunotherapy in NSCLC patients. Front. Oncol..

