# Supplementary material for: Transcriptomic Profiling of MicroRNA and Non-Coding RNA from Whole Blood of African Americans with MASLD
Source: Int J Mol Sci. 2026 Feb 9;27(4):1666. doi: 10.3390/ijms27041666 (PMC12940169; doi:10.3390/ijms27041666)
Supplement: Supplementary file 1 [file ijms-27-01666-s001.zip › ijms-4087846-supplementary.pdf]

Supplementary Table S1: Key Dysregulated Molecules–Pathways–MASLD Phenotype Associations

**Molecule**	**Fold Change Direction**	**Biological Pathway(s)**	**MASLD Phenotype Relevance**
miR-206	↑	AMPK, PPARα, MAPK	Lipid metabolism dysregulation; compensatory response to insulin resistance
miR-224-5p	↓	TGF-β/SMAD, AMPK	Hepatic fibrosis, inflammation, stellate cell activation
miR-218-5p	↓	TGF-β, Elovl5-mediated lipogenesis	Hepatic lipogenesis, fibrogenesis
miR-185-3p	↓	NF-κB, inflammatory signaling	Systemic inflammation, metabolic stress
miR-1343-5p	↓	TGF-β signaling	Fibrosis susceptibility, extracellular matrix remodeling
miR-1299	↓	Wnt/β-catenin, STAT3	Fibrogenesis, metabolic regulation
miR-193a-5p	↓	TGFB2, JNK/c-Jun	Early fibrosis, hepatic injury
LINC00963	↓	EZH2/AKT	Hepatic stellate cell activation, fibrosis
CYTOR	↑	YAP1, Hippo signaling	Fibrogenic transcriptional activation
WDFY3-AS2	↑	Wnt/β-catenin inhibition	Potential anti-fibrotic modulation

Supplementary Table S2: Fibrosis/Steatosis stages of individual MASLD Patients

**Variable**	**Discovery Cohort** (Total **n**=4**)**	**Validation Cohort (**Total **n=14)**
FibroScan**	Patient 1 – F0; Patient 2 – F2-F3; Patient 3 – F0-F1; Patient 4 – F0-F1	Patient 5 – F2; Patient 6 – F4; Patient 7 – F0-F1; Patient 8 –F0-F1; Patient 9 – F3; Patient 10 – F2; Patient 11 – F2; Patient 12 – F0-F1; Patient 13 – F0; Patient 14 – F0; Patient 15 – F0-F1; Patient 16 – F2; Patient 17 – F2; Patient 18 – F0
Steatosis Stage***	Patient 1 – S3; Patient 2 – S3; Patient 3 – S1; Patient 4 – S3	Patient 5 – S1-S2; Patient 6 – S1-S2; Patient 7 – S3; Patient 8 – S0; Patient 9 – S0; Patient 10 – S3; Patient 11 – S2; Patient 12 – S2; Patient 13 – S2-S3; Patient 14 – S0; Patient 15 – S0; Patient 16 – S3; Patient 17 – S3; Patient 18 – S0

Supplementary Table S3: Fold Change and p-Values of Key miRNAs in Discovery vs. Validation Cohorts

**miRNA ID**	**Fold Change**		**p-Value**
	Discovery Cohort	Validation Cohort	
miR-206	2.22±0.19	0.82±2.29	0.04
miR-1343-5p	-3.98±2.50	–0.74 ± 1.94	0.07
miR-224-5p	-2.65±0.52	–4.07 ± 2.24	0.04
miR-1299	-3.59±1.71	–0.42 ± 2.73	0.02
miR-193a-5p	-1.79±0.26	–0.43 ± 1.55	0.006
miR-185-3p	-2.59±1.06	–1.78 ± 1.90	0.29
